# Adaptive Coping and Caregiving Self-Efficacy Among Black Family Caregivers of Persons with Dementia

**DOI:** 10.1093/geroni/igaf122.521

**Published:** 2025-12-31

**Authors:** Sheria Robinson-Lane, Lu Qin, Florence Johnson, Ivo Dinov, Bruno Giordani

**Affiliations:** University of Michigan School of Nursing, Ann Arbor, Michigan, United States; University of Michigan, Ann Arbor, Michigan, United States; University of Michigan School of Nursing, Ann Arbor, Michigan, United States; University of Michigan School of Nursing, Ann Arbor, Michigan, United States; University of Michigan, Ann Arbor, Michigan, United States

## Abstract

Black family caregivers of persons with dementia have an increased risk of poor health, future cognitive impairment, and premature death related to stressors associated with caregiving. Understanding the relationships between adaptive coping, caregiving self-efficacy, and health can facilitate the development of resilience-focused interventions that may improve caregiver health. We surveyed 102 self-identified Black family caregivers of persons with dementia. Survey measures included the coping and adaptation processing scale (CAPS), the revised scale for caregiving self-efficacy, and the PROMIS global health scale. Analysis was completed using regression modeling. Adjusting for age, gender, marital status, employment status, income, education, and presence of chronic disease, we found that adaptive coping was positively associated with caregiver self-efficacy in managing disruptive behaviors (B = 1.57, 95% CI [.96, 2.173], p = 0.000) and controlling upsetting thoughts (B = 1.67, 95% CI [1.13, 2.22], p = 0.000). Adaptive coping was not significantly associated with mental health (i.e., emotional distress, social role satisfaction) (B=.07, 95% CI [-.28, 0.41], p = 0.707), or physical health (i.e., physical function, pain, fatigue) (B=-0.01, 95%CI [-.27, 0.24], p = 0.926). Adaptive coping strategies associated with caregiving self-efficacy and resilience theory included experience, use of planning skills, and humor. Designing caregiver support interventions that facilitate the development of effective planning skills may significantly improve caregiver mental health.

